# Ticks (Acari: Ixodidae) and tick-borne diseases in Cameroon: Current understanding and future directions for more comprehensive surveillance

**DOI:** 10.1016/j.onehlt.2024.100949

**Published:** 2024-12-09

**Authors:** Yannick Ngnindji-Youdje, Michel Lontsi-Demano, Adama Zan Diarra, Juluis Foyet, Timolèon Tchuinkam, Philippe Parola

**Affiliations:** a Aix Marseille Univ, Marseille, France; b IHU-Méditerranée Infection, 19–21 Boulevard Jean Moulin, 13005 Marseille, France; c Vector-Borne Diseases Laboratory of the Research Unit for Biology and Applied Ecology (VBID-RUBAE), Department of Animal Biology, Faculty of Science of the University of Dschang, PO Box 067, Dschang, Cameroon; d AgroEcoHealth Platform, International Institute of Tropical Agriculture, PO Box 0932, Cotonou, Benin; e Campus International IRD-UCAD Hann, Dakar 1386, Senegal

**Keywords:** Ticks, Cameroon, Distribution, Tick-borne diseases, Zoonoses

## Abstract

Despite the high burden of human and animal infectious diseases in Cameroon, implementing integrative approaches to managing and controlling arthropods and their pathogens remains challenging. Surveillance should be designed to detect diseases and provide relevant field-based data for developing and implementing effective control measures to prevent outbreaks before significant public and animal health consequences can occur. Nowadays, ticks are considered the primary vectors of animal diseases in the world, and the second vector of human diseases after mosquitoes. Knowledge of their biodiversity and distribution in any given area is a crucial step towards a better implementation of control strategies. The infections transmitted by ticks remain poorly known or underestimated in Cameroon. Despite the existence of several studies on ticks and associated pathogens, no single review to date summarises all the data available in this field in Cameroon. Following a comprehensive literature search, an inventory of the diversity and distribution of ticks, as well as the different tick-borne diseases (viral, bacteria and protozoa) found in Cameroon was prepared. To date, about 71 species, comprising ten *Amblyomma* species., eight *Hyalomma* spp., 26 *Rhipicephalus* spp., 11 *Haemaphysalis* spp.*,* seven *Ixodes* spp.*,* five *Aponomma* spp. (currently the *Bothriocroton* species), one *Dermacentor,* and four soft tick species of minimal or unknown medical and veterinary importance, namely *Argas persicus, A. arboreus, Carios vespertilionis,* and *Ogadenus brumpti* have been collected in Cameroon. Many zoonotic tick-borne diseases, such as babesiosis, theileriosis, anaplasmosis, ehrlichiosis, rickettsioses, and Q fever have been reported in the country. Knowledge about tick species and their distribution will aid in designing integrated vector management programs to monitor tick-borne diseases in Cameroon.

## 1 Introduction

Ticks are obligate blood-sucking arthropods of significant economic and sanitary importance which parasitise several classes of terrestrial vertebrate, especially mammals, birds, reptiles, and amphibians, and, accidentally, humans around the world [1,2]. Currently, almost 900 species are known and are divided into three families: the Nuttalliellidae, which is monotypic (comprising a single species *Nutattalliella namaqua*), the Ixodidae or hard ticks (including 14 genera and ∼702 species), and the Argasidae or soft ticks (including five genera and ∼200 species) [3,4]. Ticks are widely distributed around the world and can be found everywhere [5]. Different species are distributed in several biogeographical areas, both in tropical and temperate zones [6].

Approximately 10 % of the 900 currently known tick species are of significant medical and/or veterinary importance [7]. Ticks are considered the primary vectors of animal diseases in the world, and the second biggest vectors of human disease after mosquitoes [5,8,9]. While taking a blood meal, ticks can both directly and indirectly harm the host. They are known to induce severe toxic conditions such as irritation, inflammation, paralysis, allergies, abscesses, immunosuppression, anaemia, and skin damage at the biting site [10,11]. More importantly, ticks can also transmit severe infections, as they can be vector of various pathogens [7]. Of all arthropods, ticks transmit the greatest variety of pathogenic micro-organisms, including viruses, bacteria, protozoa, and helminths [8,12]. A current estimate of economic losses from ticks and tick-borne diseases globally is approximately between US$20 billion and US$30 billion per year [13].

Despite the high burden of animal and zoonotic infectious diseases in Cameroon, implementing integrative approaches to managing and controlling ticks and their pathogens remains challenging. The lack of epidemiological knowledge on ticks and tick-borne pathogens (TBPs) circulating in this country, due to the uncontrolled movement of animals (legal and illegal trade) [14] and bird migration [15], combined with a lack of expertise in the management of TBDs, limits the development of effective control strategies. Due to its geostrategic position in the central African region, Cameroon is a cosmopolitan area and plays a central role in the trade of livestock (the main hosts of ticks) both within the region and between central and West Africa [16]. Besides the movement of animals for trade purposes, transhuman pastoralism is a regional phenomenon in West and Central Africa. Live animals move from West Africa to Cameroon, especially during the dry season for nutrition and trading purposes. Unrestricted animal movements across borders have made Cameroon epidemiologically linked with both neighbouring and distant West African countries. This facilitates the spread of transboundary tick-borne diseases, such as African swine fever and, to lesser extent, lumpy skin disease, for which the vector role of ticks remains to be clearly understood [[17], [18], [19], [20]]. Cameroon displays a great diversity of agro-ecological zones (AEZs) and has an equatorial climate suitable for the establishment of ticks and tick-borne diseases [21]. Therefore, new insights into ticks and the epidemiology of TBPs are needed to address the emergence of TBDs in this area. Accurate knowledge about the diversity of TBPs circulating in a specific region is a critical step towards in implementing effective TBD prevention and control programmes.

According to literature from the 1950s and 1960s, about 53 Ixodid tick species are known from domestic and wild fauna in Cameroon [[22], [23], [24]]. The most comprehensive study dates back to 1958 [25]. Since Morel's work in 1965 on ticks in Cameroon, which revealed around 44 species, no further inventory of the general situation has been conducted [24]. However, according to numerous studies, Cameroon has a diverse tick fauna, with around 75 tick species belonging to 11 genera being recorded so far in the country [16,24,[26], [27], [28], [29], [30]]. The present study aimed to review and update information on ticks and TBPs of medical and veterinary importance in Cameroon. This information is useful in the identification of new constraints, as a first step towards research and the development of priority definitions.

## 2 Methods

### 2.1 Search method

Data on the diversity of ticks and their role in transmitting tick-borne diseases in Cameroon were gathered from published literature following the approach outlined previously [31]. Information was retrieved through searches on online bibliographic platforms such as PubMed, Google, and Google Scholar. The search utilized combinations of keywords, including: “tick fauna”, “Ixodidae tick”, “Argasidae tick”, “tick-borne diseases”, “tick-borne pathogens”, “tick-borne virus”, “tick-borne rickettsia”, “tick-borne bacteria” and “Cameroon”. Scientific publications, including books detailing tick control methods, were also reviewed. Additionally, data were collected from various sources, such as reports, books, and theses, to supplement the findings from the online databases [32].

### 2.2 Data extraction and consideration

This review primarily focused on literature and articles concerning ticks and tick-borne pathogens affecting vertebrates or mammals. Studies involving modeling or unpublished data were excluded. For each selected publication, key information was extracted and organized into a Microsoft Excel datasheet. The extracted data included the study year, methods used to identify tick species and associated pathogens, tick species investigated, study location, control methods, host characteristics, data sources, pathogen species studied, and the tick-borne diseases reported in domestic animals, wild animals, and humans in Cameroon. The compiled data were analyzed to gain insights into tick diversity and their role in pathogen transmission.

### 2.3 Tick fauna in Cameroon

**Fig. 1:**
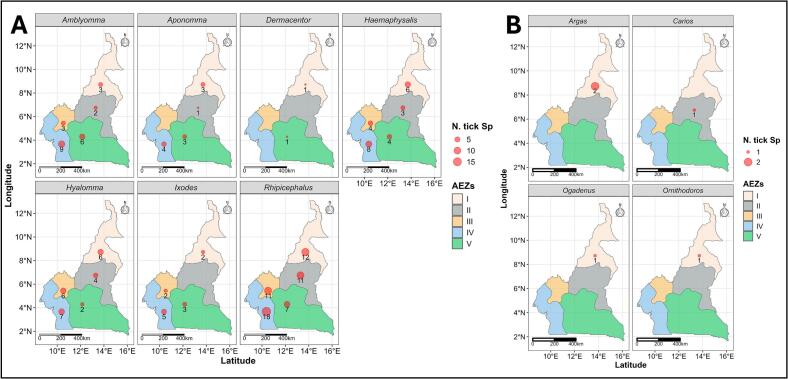
Maps showing the distribution of tick species by agro-ecological zone in Cameroon. A = Ixodidae (hard ticks), B = Argasidae (soft ticks).

Tick collection surveys conducted since 1958 have revealed a wide diversity of tick species across the five agro-ecological zones in Cameroon, [16,[26], [27], [28], [29], [30],[33], [34], [35], [36], [37], [38], [39], [40]]. In total, 73 species grouped into 11 genera have been recorded in the country to date (Fig. 1). Among these diverse species, some are of proven medical and veterinary importance, namely *Amblyomma variegatum* (*A. variegatum*), *Rhipicephalus microplus* (*R. microplus*)*, Rhipicephalus decoloratus* (*R. decoloratus*), *Rhipicephalus annulatus* (*R. annulatus*), *Rhipicephalus sanguineus* s.l. (*R. sanguineus* s.l.), *Hylomma rufipes* (*Hy. rufipes*), and *Hyalomma truncatum* (*Hy. truncatum*). The distribution of all tick species in Cameroon is available in an interactive map at the following link: https://juluis-foyet.shinyapps.io/Ticks_Distribution_Cameroon/

### 2.4 Hard tick genera of medical and veterinary importance

#### 2.4.1 *Amblyomma* species

The genus *Amblyomma* is the most important in sub-Saharan Africa, with several species playing major roles in the transmission of diseases to humans and animals, mainly livestock. This genus is composed of 130 species worldwide, including 20 species previously classified in the genus *Aponomma* [3,41]. Ticks of the *Amblyomma* genus are relatively large, with some engorged females growing up to 1.5 cm in length. They are characterized by dorsal integumentary ornamentation, the presence of eyes, and a well-developed mouthpart. These ticks exhibit a trixene life cycle, requiring parasitic phases on three distinct hosts, and show telotropic behavior, meaning their host preferences vary across developmental stages. *Amblyomma* ticks serve as both vectors and reservoirs for a variety of pathogens that can affect animals and humans [5,41,42]. According to recent records, ten species have been reported in Cameroon [16,29,30,34,43,44] (Additional file Table S1). Their distribution varies across ecological zones. *Amblyomma variegatum* is the commonest and most widely distributed ticks on livestock in Cameroon and is considered as major vector of ehrlichiosis in animals and tick typhus in humans in some African countries. Although native to southern African countries, *Amblyomma hebraeum* has also been reported in Cameroon, particularly in the West and North-West regions [25,44,45].

#### 2.4.2 *Hyalomma* species

*Hyalomma* is a genus comprising 27 species worldwide [3]. Ticks of the *Hyalomma* genus are notable for their elongated palps, which are at least twice as long as they are wide. Their distinct eyes are situated in sockets near the postero-lateral edges of the scutum. These ticks are generally medium to large in size, with long mouthparts, and lack dorsal ornamentation. While the three-host life cycle is most common in this genus, some species follow one- or two-host cycles. Additionally, certain three-host species possess a unique facultative ability among ixodid ticks to develop using one- or two-host cycles. The genus *Hyalomma* contains ticks of veterinary and public health importance [3,6,42] To date, eight known species and a number of unidentified ticks have been collected in Cameroon [29,30,[34], [35], [36], [37],^39^,[46], [47], [48]] (Additional file Table S2). Species such as *Hy. nitidum, Hy. rufipes,* and *Hy. truncatum* are the most widely distributed ticks across the country [27,29,30,37,43,46,49,50]. *Hyalomma truncatum* is found predominantly in Africa, south of the Sahara, where it is the commonest *Hyalomma*. In southern Africa, *Hy. rufipes* is the most important vector of the Crimean-Congo haemorrhagic fever virus in humans [6].

#### 2.4.3 *Rhipicephalus* species

This genus comprises 82 species worldwide, including five species from the former genus *Boophilus*, which is still considered valid by some authors [3]. Ticks of the *Rhipicephalus* genus are typically small, inornate, and reddish-brown, with relatively short mouthparts and slight sexual dimorphism. A distinctive feature is the hexagonal shape of the basis capituli, easily observed in a dorsal view. These ticks predominantly parasitize mammals, while immature stages occasionally infest birds or reptiles [6,51]. To date, 26 species, including four species from the former genus *Boophilus* and some unidentified *Rhipicephalus* spp., have been collected in Cameroon [16,26,29,30,[37], [38], [39],^43^,[52], [53], [54]] (Additional file Table S3). In this article we will use the term *Rhipicephalus* for this genus. Six species are by far the most mentioned or studied, and widely distributed in the country. These include *R. annulatus, R. decoloratus, R. microplus, R. sanguineus* s.l.*, R. lunulatus,* and *R. longus. Rhipicephalus microplus,* one of the most significant cattle ticks in the world, was reported for the first time in Cameroon in 2019 [55]. The findings of this study indicate that the newly introduced tick has largely supplanted native *Rhipicephalus* species on cattle in the study areas, with *R. decoloratus* being the most affected. Since the initial detection of *R. microplus*, its range has expanded to encompass nearly the entire country, covering four out of the five agro-ecological zones [27,29,30,37,55]. This invasive Asian cattle tick is believed to have entered the country via livestock movements associated with trade [19], particularly through exchanges with Nigeria. Nigeria, a West African nation, has harbored this tick species since 2007, following its introduction from Côte d'Ivoire [56]. From Nigeria, *R. microplus* continues to spread, further establishing itself across other countries in the western and Central African region [32,[57], [58], [59]].

#### 2.4.4 *Haemaphysalis* species

The genus *Haemaphysalis* is composed of 166 species [3]. *Haemaphysalis* tick is recognised by the pronounced lateral projection of palpal segment 2 in most species, which extends well beyond the basis capituli. These small ticks lack eyes. *Haemaphysalis* species parasitize birds and mammals in most regions of the world [3,60]. Thus so far, 11 known species and several unidentified *Haemaphysalis* spp. ticks have been collected in Cameroon [30,33,34,40,43,[61], [62], [63], [64], [65], [66]] (Additional file Table S4). *Haemaphysalis leachi,* also known as the “yellow dog” tick, is by far the most frequently mentioned and studied species and is the most widely distributed species in the country [29,30,35,43,53,64]. Alongside *R. sanguineus* s.l., this tick has adapted to feed on domestic dogs in tropical and subtropical areas and is found mainly on domestic dogs in sub-Saharan Africa [6]. Species such as *Ha. aciculifer, Ha. hoodi*, and *Ha. parmata* are the second most widely distributed ticks of the genus in Cameroon [26,30,43,50].

### 2.5 Other hard tick genera of unknown or minimal medical and veterinary importance

#### 2.5.1 *Ixodes* species

The genus *Ixodes* has the highest number of species worldwide with up to 243 species [3]. A total of seven species have been reported in Cameroun [29,30,36,50] (Additional file Table S5). The most represented species are *I. rasus* and *I. cumulatimpunctatus.* Although these species are associated with livestock, their role as disease vectors in Cameroon has not yet been described.

Due to a lack of isolation and few attempts to do so, the *Aponomma* (currently the *Bothriocroton* species) and *Dermacentor* genera have not been implicated as disease vectors in Cameroon, and their relationship to disease is unknown. It is important to note that species of these two genera have been reported much more in the northern, central, and coastal parts of the country than elsewhere [24,30].

### 2.6 Soft tick genera of unknown or minimal medical and veterinary importance

Although the taxonomy of soft ticks is still the subject of much controversy, particularly at genus level, this family includes 193 species worldwide [3].

According to some authors (Morel and Mouchet, 1965; Guglielmone et al., 2010), there are between four and ten genera, the main ones being *Antricola, Argas, Nothoaspis, Ornithodoros,* and *Otobius* [3,24]. In Cameroon, researchers have identified four species of Argasidae, distributed across three distinct genera:: *Argas percicus, A. arboreus, Carios vespertilionis,* and *Ogaadenus brumpti* [43] (Additional file Table S6). These species have not been implicated as disease vectors in Cameroon, and their relationship to disease is unknown. However, in drier Sahelian habitats of West Africa, such as Senegal, *Ornithodoros sonrai* colonise rodent burrows in proximity to and within human habitations and are responsible for the occurrence of tick-borne human relapsing fever borreliosis. This species of *Ornithodoros* tick is widespread in West Africa, with 97 % of the villages in some regions being infested [[67], [68], [69]]. It is important to note that since Morel and Mouchet in 1965, species of these genera have only been collected in the northern part of the country.

### 2.7 Tick-borne diseases and pathogens reported in Cameroon

Many tick-borne diseases, such as protozoan diseases (babesiosis and theileriosis), bacterial diseases (anaplasmosis, ehrlichiosis, and Rickettsiosis) and viral diseases (Crimean-Congo haemorrhagic fever) have been reported in Cameroon [[70], [71], [72], [73]]. Several studies have shown that ticks can be infected with bacteria, viruses, and protozoa. The diseases they transmit cause significant economic losses, primarily in the livestock industry, and mainly affect tropical and subtropical countries, where ticks constitute one of the main difficulties for the development of the livestock breeding industry [8].

In Cameroon, ticks transmit diseases to humans and livestock, and the most significant genera are *Amblyomma, Rhipicephalus, Hyalomma*, and *Haemaphysalis*. The main tick-borne diseases of veterinary importance in Africa are anaplasmosis, heartwater, babesiosis, and theileriosis [6,8]. However, in this review we report the following major tick-borne diseases of medical and veterinary importance in Cameroon: protozoa diseases (babesiosis and theileriosis), bacterial diseases (anaplasmosis, ehrlichiosis, African tick-bite fever, spotted fever rickettsioses, and borreliosis), and viral diseases (Crimean-Congo haemorrhagic fever) (Table 1).

**Table 1:** Tick-borne diseases, such as protozoan (babesiosis and theileriosis), bacterial (anaplasmosis, ehrlichiosis, and rickettsiosis) and, viral diseases (Crimean-Congo haemorrhagic fever) reported in Cameroon.

Diseases	Infectious agents	Infected hosts detected	Vectors	Locations	AEZs	Reference
**Protozoa**						
Bovine Babesiosis (cattle fever)	*B. bigemina B. bovis*	Cattle	*R. microplus, R. decoloratus, R. annulatus*	Bambili, Bambui, Dschang, Fokoue, FongoTongo, Nkong-Ni	III	[27,74,77]
Mediterranean theileriosis	*Th. annulata*	Cattle	*Hyalomma* spp.	Far north region	I	[80]
East Coast Fever	*Th. parva*	Cattle	*Rhipicephalus* spp.	Mayo-Louti, Benoué, Mayo-Banyo, Djérem, Mbere, Vina, Faro-et-Deo, Kadey, Noun, Haute Sanaga	I, II, III, V	[82]

Theileriosis	*Th. velifera*	Cattle	*Am. variegatum*	Far north region	I	[80]
	*Th. mutans*	Cattle	*Rhipicephalus* spp.	Far north region	I	[80,82]

**Bacterial**						
Bovine Anaplasmosis	*A. marginale* *A. centale*	Cattle	*R. microplus* *Rhipicephalus* spp.	Far north region, Bambili, Bambui, Dschang	I, III,	[73,74,80]
Anaplasmosis (Infectious trombocytopaenia)	*A. platys*	Cattle	*Rhipicephalus* spp.	Far north region, Dschang	I	[73,74,80]
Anaplasmosis	*A. sp Hadesa*	Cattle		Far north region	I	[80]
Heartwater	*E. ruminantium*	Cattle; Sheep and Goats	*Am. variegatum*	Garoua, Mindif (Kaélé), Yagoua, Badjari, Lougguere and Mora, Buea, Dumbo, Dschang	I, III, IV	[46,73,80,87,88]
Canine monocytic ehrlichiosis	*E. canis*	Cattle, Dogs	*R. sanguineus* s.l.	Far north region, Buea, Douala, Limbé	I, IV	[62,63,80,90]
African tick-borne fever (ATBF)	*R. africae*	Cattle, Human	*Am. variegatum*	Garoua, Mudemba, Mouanko, Lomié, Massagan, Njikwa, Nyambisan, Sobia, Buea, Limbé, Muyuka, Tiko; Ngaoundéré, Dschang, Nkong-Ni, Kouptamo, Massangam, Koutaba	I, III, IV, V	[70,73,80,95,96]
Human granulocytic Ehrlichiosis	*E. ewingii*	Dogs	*R. sanguineus* s.l.	Buea, Douala	IV	[62,63]
Human monocytic Ehrlichiosis	*E. chaffeensis*	Dogs, Human	*R. sanguineus* s.l.	Buea, Douala, Tiko, Limbé	IV	[62,63,90]
Rickettsiosis	*R. aeschlimanni*	Cattle	*Hy. rufipes, Hy. truncatum*	Ngaoundéré, Dschang	II	[73,96]
Rickettsiosis	*R. sibirica*	Cattle	*Hy. truncatum*	Ngaoundéré	II	[96]
Rickettsiosis	*R. massiliae*	Cattle	*R. lunulatus*	Ngaoundéré, Dschang, Nkong-Ni, Kouptamo, Massangam, Koutaba	II	[73,96]
Rickettsiosis	*Candidatus R. barbariae*	Cattle, Human	*Am. variegatum, Hy. rufipes, R. lunulatus, Hy. truncatum*	Ngaoundéré, Dschang	II	[73,96]
Coxiellosis (Q fever)	*C. burnetii*	Cattle	*Hy. truncatum, Hy. rufipes*	Ngaoundéré, Dschang, Nkong-Ni, Kouptamo, Massangam, Koutaba	II, III	[73,96]

***Virus***						
Crimean-Congo Haemorrhagic Fever	*CCHF Virus*	Human	*Hy. marginatum*	Abomg Mbang, Lomié, Messok, Mindourou	V	[71]
Non-pathogenic	*Bhanja arbovirus (Yak-10)*	Cattle	*R. decoloratus*	Garoua	I	[109]
	*Dugbe virus*	Cattle	*Am. variegatum*	Yaoudé, Edéa, Obala	IV, V	[34]

### 2.8 Protozoan diseases

#### 2.8.1 Babesiosis

Babesiosis, also known as piroplasmosis, is a disease caused by protozoan parasites of the genus *Babesia* [74]. The protozoa of the genus *Babesia* are strictly intra-erythrocytic parasites transmitted by hard ticks of the family Ixodidae. For “large” *Babesia* (> 2.5 μm), transovarian transmission from the infected female tick to her offspring is possible. This is why tick larvae and nymphs can be a source of parasites for mammals. Parasites of the genus *Babesia* are generally very specific to their hosts, whether it is for tick vector species or mammalian hosts [75]. These pathogens are transmitted to cattle by ticks of the genera *Rhipicephalus* and cause the disease known as bovine babesiosis. Other names of the disease are Cattle Tick Fever, Texas fever, Redwater and Splenetic Fever. The prevalence of *Babesia bovis*, the more pathogenic species causing babesiosis in cattle, was reported as 47.3 % in Bambili and 31.1 % in Bambui, both located in agro-ecological Zone III [74]. Additionally, the prevalence of *B. bovis* and *B. bigemina* has been assessed in cattle within the Menoua [76] and Vina divisions [77]. However, it is important to note that *Babesia bovis* is exclusively transmitted by *R. annulatus* and *R. microplus*, whereas *Babesia bigemina* is primarily transmitted by *R. decoloratus* and other tick species.

#### 2.8.2 Theilerioses

Theileriosis is a tropical haemoprotozoal disease caused by obligate intracellular protozoan parasites of the genus *Theileria* (phylum Apicomplexa, order Piroplasmida, family Theileriidae). It is transmitted through the bite of hard ticks, with the most significant genera being *Amblyomma*, *Haemaphysalis*, *Hyalomma*, and *Rhipicephalus* [78]. They are most closely related to Babesia, from which they differ by having a developmental stage in leukocytes prior to infection of erythrocytes. This agent infects both wild and domestic Bovidae throughout the world and some species infect small ruminants. There are several identified *Theileria* spp. that infect hosts around the world, particularly cattle. The most pathogenic and economically significant of them are *Theileria parva* which causes East Coast Fever (ECF), *Theileria annulata*, which causes tropical theileriosis (TT) and Mediterranean theileriosis, and *Theileria orientalis* (*T. orientalis*/*buffeli* group), which causes Oriental theileriosis (OT) and Theileria-associated bovine anaemia (TABA) [79]. The ECF of ruminants (cattle) is a serious problem in Cameroun, notably in AEZ I, II, III and V. The causative agents in these areas are *Theileria mutans, T. parva,* and *Theileria velifera.* [[80], [81], [82]]. According to Abanda (2019a) *T. mutans* and *T. velifera* had a high incidence in northern Cameroon with a prevalence of 90.3 % and 77.4 %, respectively [80]. Detection by sequencing produced unknown *Theileria* sp. in three cases, *T. velifera* in one case, *T. mutans* in 17 cases, and *T. mutans* co-infected with *T. velifera* in three cases. In 85.7 % (24/28) of the cases, *T. mutans* was found in co-infection with *T. velifera,* which is significantly higher than that recorded by Sanger sequencing of the PCR product (13.6 %; 3/22). Both *T. annulata* and *T. parva* were not found either by sequencing or by LCD array [81], and the overall prevalence of *Theileria* spp. was 57.3 % for *T. mutans*, 2.7 % for *T. velifera*, 0.5 % for *Theileria* sp., and 18.4 % for *Theileria* sp., identified only to the genus level [80] depending on the identification technique used. The seroprevalence of *T. parva* and *T. mutans* was assessed using ELISA tests. The overall mean antibody prevalence was 22.75 % for *T. parva*. The average serum antibody prevalence of *T. parva* was relatively constant in all AEZs. It was 21.42 %, 24.28 %, 19.64 %, 21.42 %, and 22.77 % for AEZ I, II, III, IV and AEZ V, respectively [82]. It is important to note that this was the first time *Theileria parva* had been detected in cattle in Cameroon. The most important vector for *T. parva* is *Rhipicephalus appendiculatus, Ripicephalus zambeziensis* in southern Africa, and *Rhipicephalus duttoni* in Angola, which can also spread ECF.

### 2.9 Bacterial diseases

#### 2.9.1 Anaplasmosis

Anaplasmosis is a tick-borne disease caused by bacterium of the genus *Anaplasma.* There are several forms of anaplasmosis notably human anaplasmosis, which is also known as human granulocytic anaplasmosis (formerly known as human granulocytic ehrlichiosis), caused by *Anaplasma phagocytophilum*. This is an intracellular bacterium transmitted by *Ixodes* ticks in the United States and Europe [83]. Other forms of anaplasmosis are known to affect animals, notably cattle in Cameroon, and are caused by four species of *Anaplasma* [74,80,81]. Bovine anaplasmosis can be caused by *A. phagocytophilum, A. marginale, A. centrale* and *A. bovis*. [84]. This infection is known to be one of the most economically significant diseases in the cattle industry, both in Cameroon and in several other African countries [85]. Another name for the disease is gall sickness. Ixodid ticks of the genera *Rhipicephalus* are the mains vectors in Cameroon [6]. Conventional PCR with generic primers was used to identify groups of tick-borne pathogens in cattle breeds from northern Cameroon (AEZ I). Sanger sequencing of a subset of positively tested samples revealed the presence of *A. centrale* (10.9 %), *A. marginale* (30.7 %), *A. platys* (51.1 %), and *Anaplasma* sp. ‘Hadesa’ (10.9 %). In total, four species of *Anaplasma* were tested on a single LCD array [80,81]. Antibodies against *A. centrale* and *A. marginale* have been detected in the blood of cattle in the Menoua [76]. In ticks, the DNA of *A. centrale* and *A. marginale* was found in *R. microplus* and *Ha. leachi* from the western highlands of Cameroon [73]. To the best of our knowledge, no cases of human anaplasmosis have yet been detected in Cameroon.

#### 2.9.2 Ehrlichiosis

Ehrlichiosis is a disease that infect a wide range of mammals and is caused mainly by a bacterium of the genera *Ehrlichia. Ehrlichia* are gram-negative, pleomorphic, obligate intracellular bacteria that comprise five species: *E. canis*, *E. chaffeensis*, *E. ewingii*, *E. muris,* and *E. ruminantium* (formerly *Cowdria ruminantium*) [86]. In public health, there are currently two recognised diseases caused by *Ehrlichia* species: human monocytic ehrlichiosis (HME), caused by *E. chaffeensis*, and human granulocytic ehrlichiosis (HGE), due to *E. ewingii.* Several species of *Ehrlichia* have been reported in Cameroon, mainly *Ehrlichia ruminantium* since 1997, *E. canis*, *E. chaffeensis* and *E. ewingii.,* mainly transmitted by species of *Amblyomma* and *Rhipicephalus* spp. [46,62,63,73,80,81,[87], [88], [89], [90]]*.*The main causative agent of animal ehrlichiosis in Cameroon is *E. ruminantium* in cattle, but all ruminants can carry the causative agent, some without showing any apparent clinical signs of disease. Bovine ehrlichiosis is also known as “heartwater”*.* The principal vector is *Am. variegatum,* but the causative agent has been detected in other ticks, notably *R. sanguineus* [63]*. Ehrlichia* spp. also causes different diseases in Cameroon livestock, including sheep and goats, caused by ovine ehrlichiosis. This bacterium also affects dogs and causes canine ehrlichiosis. Recent studies conducted on cattle in northern Cameroon have shown that a single infection of *E. canis* and *E. ruminantium* was found in Mayo Rey and Faro-et-Deo in AEZ I. According to the proportions of the identified *Ehrlichia* spp. in all study sites, the prevalence was 0.5 % for *E. ruminantium*, and 0.5 % for *E. canis* [80]. By comparing two diagnostic techniques *Ehrlichia* species were detected in 17 (54.8 %) of the screened samples using the Low-Cost Density Array, a proportion significantly higher than the 3.2 % prevalence identified through Sanger sequencing. Among the unsuccessfully sequenced samples screened under the Low-Cost Density Array, *E. ruminantium* was found in co-infection with another bacterial agent (*A. centrale* and *A. marginale*). *E. canis* was found by sequencing and hybridised by its specific probe on the array in only one sample, however this was below the threshold of 2000 pixel values [81].

Another study in Dumbo (AEZ III) and Buea (AEZ IV) was performed, which revealed serologic and microscopic evidence of the presence of heartwater in ruminants in Cameroon. Moreover, PCR amplification of the 279 bp fragment of the pCS20 region detected *E. ruminantium* DNA in 142 of the 500 ticks (28.4 %), with a higher infection rate (40.9 %) being observed in ticks from Dumbo, and 24.7 % of ticks collected from cattle in Buea. This report represents the first molecular evidence of *E. ruminantium* infection in *A. variegatum* ticks in Cameroon and suggests possible exposure of cattle to this pathogen in our environment. [87,88].

#### 2.9.3 Tick-borne relapsing fever borreliosis

Tick-borne relapsing fever (TBRF) is caused by several species of spiral-shaped bacteria (spirochaetes) that are transmitted to humans or animals through the bite of infected ticks. In humans, the disease is characterized by multiple recurrences of fever, headache, myalgia, and arthralgia caused by bacteria of the genus *Borrelia.* In Africa, tick-borne relapsing fevers are caused by four principal cultured species, *B. crocidurae*, *B. duttonii, B. hispanica,* and *B. percica* [91,92].

Initially, the disease was found mainly in East Africa, where the name East African tick fever (*B. duttonii*) originated, responsible for high morbidity and mortality, particularly among children and pregnant women and transmitted by *Ornithodoros moubata* s.l. [67,93]. It was then found in West Africa, where it is also known as recurrent fever (*B. crocidurae*), transmitted by *Ornithodoros sonria*. Finally it was found in North Africa, where this relapsing fever (*B. hispanica*) is transmitted by *Ornithodoros erraticus* and in Egypt, *B. percica* transmitted by *Ornithodoros tholozani* [67,93]. There is also the presence of *B. tillae* in South Africa, transmitted by *Ornithodoros zumpti*, whose pathogenicity is not clearly defined. Prior to 2018, no bacteria of the *Borrelia* genus had ever been detected in Cameroon. It was in 2019 that Abanda et al., in their investigations in the northern part of the country, reported a case of *Borrelia* infection in cattle for the first time [80]. *Borrelia* pathogens were identified in all northern regions studied, with the Adamawa region having a significantly higher prevalence (52.4 %). The only species identified by sequencing was *B. theileri,* with an overall prevalence of 17.9 % [80]. Another study showed a prevalence of 1.3 % of *B. theileri* in *R. microplus* in the Menoua division, in the western region of Cameroon [73].

#### 2.9.4 Spotted fever group rickettsioses

Tick-borne rickettsioses are infectious diseases caused by members of the family Rickettsiaceae, and the spotted fever group of the genus *Rickettsia*. About twenty species of the SFG are transmitted by ticks [94]. To date, at least five validated SFG rickettsia have been detected in ticks, animals, and humans in Cameroon, including *R. africae,* the agent of African tick-bite fever (ATBF)*, R. aeschlimannii, R. sibirica mongolitimonae,* and *R. massiliae* [73,80,95,96]. The clinical signs of tick-borne spotted fever group rickettsioses vary from species to species mainly as eschar at the tick bite site, rash (macular, maculopapular, or vesicular), sometimes lymphangitis, fever of unknown origin and possible seriousness with neurological signs, multi-organ deficiencies, and even death [94].

African tick-bite fever is an emerging infectious disease which is endemic in sub-Saharan Africa. It is caused by the spotted fever group, *R. africae,* and is transmitted by ticks of the genus *Amblyomma* [97]. One study showed that *R. africae* was identified in seven of 118 patients (6 %) with acute fevers of unknown aetiology proven not to be malaria or typhoid fever from clinics along the coastal region of Cameroon [70]. In addition, serum samples of 32 % of the 234 acutely febrile patients at clinics in Tiko and Buea contained immunoglobulin M antibodies reactive with *R. africae* by immunofluorescence assay and were reactive with outer membrane proteins A and B of *R. africae* by immunoblotting [98]. A high *R. africae* seropositivity rate (28 %) was detected in indigenous populations in rural areas of Cameroon. This high seroprevalence rate was linked to the presence of cattle, the preferred host of *Am. variegatum*, and to lowland rainforest habitats, ideal for the behavior of this tick species [95]. DNA of *R africae* was detected in both cattle blood samples and tick samples, including *A. variegatum, Hy. truncatum,* and *R. microplus* [73,80,96]*.*

*R. aeschlimannii* is a recognised human pathogen causing spotted fever and has been detected in different countries in sub-Saharan Africa [94]. In Cameroon, *R. aeschlimannii* was identified in *Hy. rufipes* and of *Hy. truncatum.* It was also found in 2.2 % of *R. sanguineus* sl. [73,96]. However, this tick species is not known to be a competent vector of *R. aeschlimannii*. Co-feeding could therefore be the cause of infection of this tick species, as discussed above.

*Rickettsia massiliae* is a pathogenic rickettsia that is associated with *Rhipicephalus* spp. ticks. It has been described as a human pathogen in Europe and South America, but there have been no reports of human infections in Africa [94]. The first finding of this micro-organism in tick species in Cameroon was in a study conducted in Adamawa region [96]. Another finding of *R. massiliae* from *R. lunulatus* (5.6 %) and *Rh. muhsamae* (10 %) ticks was in the western highlands area of the country [73].

#### 2.9.5 Q fever

*Coxiella burnetii*, a strict intracellular gram-negative bacterium, is responsible for Q fever affecting humans and a variety of animals first described in Australia in 1937 [99]. Q fever can be found worldwide, but the epidemiological features of this disease vary according to the geographic area considered, including situations where it is endemic or hyperendemic, and the occurrence of large epidemic outbreaks [100]. Although Q fever is far more frequently airborne, at least seven hard and soft tick species, including *Hyalomma* spp., have formally been shown to be competent vectors of *C. burnetii* [101]*.* In 2022, for the first time in Cameroon, *C. burnetii* was detected in tick species at the rate of 12 %, 6.3 %, and 0.3 % in *Hy. truncatum*, *Hy*. *Rufipes,* and *R. sanguineus sl.,* respectively [73]. One study showed that in Cameroon, 9 % of community-acquired pneumonia in those aged >15 tested positive for *C. burnetii* [102].

### 2.10 Viral diseases

#### 2.10.1 Crimean-Congo haemorrhagic fever

Crimean-Congo haemorrhagic fever (CCHF) is an emerging vector-borne zoonotic disease of increasing importance caused by an *Orthonairovirus,* belonging to the Nairoviridae family. This virus is maintained in nature through an enzootic tick-vertebrate transmission cycle comprising a large panel of vertebrate hosts in which the presence of the virus is asymptomatic [103]. The primary vectors of the disease are ticks of the genus *Hyalomma,* although in Saharan Africa the genera *Rhipicephalus*, *Dermacentor*, *Amblyomma*, *Haemaphysalis,* and *Ixodes* have been found to be naturally infected by the virus [104]. The first serological study on CCHF virus among urban populations in Cameroon was conducted in 1988 yielding negative results [105]. Subsequently, Gonzalez et al. (1989) reported a seroprevalence rate of 0.22 % in the populations of Nkongsamba, Mora, and Maroua [106]. A later study by Sadeuh-Mba et al. (2018) observed a seroprevalence rate of 4.4 % among pygmies in the forests of Cameroon's eastern region, where hunting is the primary economic activity [71]. The most recent study, conducted in two livestock markets in Yaoundé, revealed an overall CCHFV seroprevalence of 61.77 % across all animals. The highest rates were observed in cattle (433/441, 98.18 %), followed by sheep (23/147, 15.65 %) and goats (11/168, 6.55 %), with a statistically significant difference (*p*-value <0.0001) [107]. It should be noted that this virus has not yet been isolated from a vector in Cameroon.

Although it has not been described as a disease-causing agent, the Dugbe virus has been isolated from *Am. variegatum* ticks collected from domestic cattle in Cameroon [34]. The Nairovirus Dugbe (Bunyaviridae family) is transmitted by *Am. variegatum* [108]. In addition, a strain of virus designated YaK-10 was isolated from *R. decoloratus* ticks collected in March 1971 from cattle pastured in savanna near Garoy, by the intracerebral inoculation of suckling mice at the Pasteur Institute in Cameroun [109]. Studies of the antigenic properties of this virus and neutralisation tests showed it to be identical with Dakar D-9540 VIRUS (Bhanja) from Senegal [110]. Despite these findings, the study of tick-borne viruses remains a largely unexplored area of research in Cameroon.

#### 2.10.2 African swine fever

African Swine Fever (ASF) is a highly contagious and often fatal viral disease affecting pigs, caused by the African Swine Fever Virus (ASFV), the only member of the Asfarviridae family. The disease poses enormous problems to the pork industry with pig mortality ranging from 30 % to 100 %, depending on the virulence of the circulating virus [111,112]. African swine fever virus (ASFV), which is a transboundary animal disease can be transmitted by direct or indirect contact with infected pigs, as well as by soft ticks of the genus *Ornithodoros*. This includes *Ornithodoros moubata* in Africa, and *O. erraticus* in Europe, which serve to transmit the virus to wild and feral suids, as well as serve as a reservoir of the virus [20]. Cameroon, located in Central Africa is one of the countries in which the African swine fever virus (ASFV) has been endemic since its first outbreak in 1982, which resulted in the death of 80 % of the pig population [113]. To date, no ASFV-carrying ticks have been identified in the country, despite the ongoing circulation of the virus. The absence of tick vectors suggests that ASF transmission in Cameroon occurs primarily through direct contact between infected and healthy pigs, or indirectly via pork products and human activities within the pig industry [113]. In West Africa, limited data on ASF exists, with research mostly concentrated in Côte d'Ivoire, Senegal, and Nigeria. ASFV DNA has only been detected in ticks of the species *O. sonrai*, which were collected from pigsties and warthog burrows in Senegal [32].

#### 2.10.3 Emerging tools for tick identification

Correct identification of arthropods and characterisation of their repertoires of pathogens are crucial steps in assessing the risk of transmission of vector-borne diseases. The oldest and most widely used method for identifying ticks remains morphological identification. However, this technique sometimes gives dubious results, which can be attributed to the difficulty of using identification keys, the quality of specimens (damaged), or a lack of expertise in entomology [114,115]. Molecular biology approaches (PCR, sequencing) have been developed for several decades to identify these arthropods by amplifying different DNA fragments [116,117]. Although this method requires expertise and an appropriate molecular biology platform, which limits its use in large-scale studies, it is reputed to be effective and reliable.

Over the last two decades, new methods have been developed for the identification of biomolecules, including proteins, and have been applied to arthropods, with the development of a multiplex technique using mass spectrometry coupled with a matrix-assisted laser desorption/ionisation time-of-flight analyser (MALDI-TOF MS). The principle is to measure the time of flight of a sample after it has been ionised by laser beams and accelerated in a flight tube according to its mass-to-charge ratio. This generates spectra representing a specific fingerprint signature of the sample [115]. From its beginnings in chemistry to the field of microbiology, the effectiveness of this tool was subsequently proven in the fields of medical and veterinary entomology and is now used routinely not only to identify numerous arthropod species and determine the origin of their blood meal, but also to determine their infectious status [118]. In Cameroon, this approach has recently been successfully used for the first time to identify tick species and bedbugs [29,119]. Despite some constraints, in particular the conditions for preserving specimens and the choice of body part to be used, MALDI-TOF MS is a competitive method in terms of cost, experimental and analysis time, and accuracy for identifying ticks at species complex level (example of tropical and temperate lineage of *R. sanguineus* sl.) and even at species level.

#### 2.10.4 Policy direction on ticks and tick-borne diseases in Cameroon

Ticks and tick-borne diseases represent a growing public health challenge in Cameroon, affecting human and animal health, as well as the economy, particularly in livestock farming. To meet this challenge, we need to focus on several strategic areas: strengthening epidemiological surveillance and research to map ticks and develop appropriate local solutions, raising awareness among rural populations and training agricultural and veterinary professionals in prevention methods, promoting tick control and environmental management techniques, strengthening veterinary and human health infrastructures, establishing a legislative framework and regional partnerships for sustainable management, and integrating local communities in the development of solutions. A “One Health” approach, integrating the interconnections between human, animal and environmental health, is essential for a national plan to reduce the impact of tick-borne diseases.

## 3 Conclusion

This review summarises and updates information on tick species of medical and veterinary interest and highlights their associated micro-organism. Tick-borne diseases are generally underestimated in Cameroon, even though multiple entomological surveys have been conducted in the country. The review reveals the great diversity of the Cameroonian tick fauna, with up to 73 species recorded, of which *R. microplus R. decoloratus R. lunulatus,* and *A. variegatum* were the most prevalent and widely distributed. Many of these species are vectors of human and animal pathogens. The study also showed that many zoonotic tick-borne diseases, such as babesiosis, theileriosis, anaplasmosis, ehrlichiosis, rickettsiosis, and Q fever have been reported. Information on the tick species and their distribution will be useful for the development of integrated vector management programmes for the surveillance and elimination of tick-borne diseases in Cameroon. Future studies on wild animals must be carried out in addition to livestock (particularly cattle) because livestock are most often in contact with these wild animals in pastures during periods of transhumance or when searching for pastures. In addition, diagnostic methods need updating. Morphological identification must be supplemented with molecular and spectral analyses to optimise the results.

## Abbreviations

**Table:** 

ASFV	African swine fever virus
ATBF	African tick-bite fever
SFG	Spotted fever group
TBRF	Tick-borne relapsing fever
TG	Typhus group
TBPs	Tick-borne pathogens
TBDs	Tick-borne diseases
AEZs	Agro-ecological zones
ECF	East coast fever
LCD	Low-cost density
TABTA	Theileria-associated bovine anaemia.
TT	Tropical theileriosis
OT	Oriental theileriosis
CCHF	Crimean-Congo haemorrhagic fever
PCR	Polymerase Chain Reaction

## Ethics approval and consent to participate

Not applicable.

## Consent for publication

Not applicable.

## Funding

This study was supported by the Institut Hospitalo-Universitaire (IHU) Méditerranée Infection, the National Research Agency under the “Investissements d'avenir” programme, reference ANR-10-IAHU-03, the Région Provence Alpes Côte d'Azur and European ERDF PRIMI funding. YNY received a grant of PhD scholarship from IHU Méditerranée Infection. The funders had no role in study design, data collection and analysis, decision to publish, or preparation of the manuscript.

## CRediT authorship contribution statement

**Yannick Ngnindji-Youdje:** Writing – review & editing, Writing – original draft, Software, Methodology, Investigation, Formal analysis, Data curation, Conceptualization. **Michel Lontsi-Demano:** Writing – review & editing, Software, Methodology, Formal analysis, Data curation, Conceptualization. **Adama Zan Diarra:** Writing – review & editing, Methodology, Investigation, Formal analysis, Data curation. **Juluis Foyet:** Software, Formal analysis, Data curation. **Timolèon Tchuinkam:** Writing – review & editing, Supervision, Methodology, Conceptualization. **Philippe Parola:** Writing – review & editing, Validation, Supervision, Funding acquisition, Conceptualization.

## Declaration of competing interest

The authors declare that they have no known competing financial interests or personal relationships that could have appeared to influence the work reported in this paper.

## Data Availability

No data was used for the research described in the article.
