# Supplementary material for: Ticks (Acari: Ixodidae) and tick-borne diseases in Cameroon: Current understanding and future directions for more comprehensive surveillance
Source: One Health. 2024 Dec 9;20:100949. doi: 10.1016/j.onehlt.2024.100949 (PMC11733189; doi:10.1016/j.onehlt.2024.100949)
Supplement: Supplementary material [file mmc1.docx]

**Table S1:** *Amblyomma* ticks (10 species), associated hosts, collection locality, region and and Agro-Ecological Zones

| **Ticks Genus** | **Tick species** | **Hosts** | **Location** | **Region** | **AEZs** | **References** |
| --- | --- | --- | --- | --- | --- | --- |
| *Amblyomma* | *Am. compressum (*formerly cuneatum*)* | White-bellied pangolins, Warthogs, Nile monitors, African brush-tailed porcupines, Monkeys, African golden cat, Antelopes, African civets | Ebolowa, Mfou, Bipindi, Lolodorf, Sikoula, Yaoundé, Lomié, Ebogho, Kribri, Yokadouma, Bibundi, Manfé, | East, South, Southwest | IV, V | [30,43] |
|  | *Am. eburneum* | Cattle | Yaounde, Edéa, Obala | Centre, Littoral | IV, V | [34] |
|  | *Am. flavomaculatum* | Nile monitors, Four-toed hedgehogs, White-bellied pangolins, Monkey, Warthog | Kaelé, Ebolowa | Far North, East | I, IV | [30,43] |
|  | *Am. hebraeum* | Cattle, Horses | Banso, Fundong, Acha, Fongo-Tongo, Fokoué, Dschang, Bangourain, Foumban, Koutaba, Bangangte | West, Northwest, | III | [25,44,45] |
|  | *Am. nuttalli* | Turtle, Bird, Porcupine, Guinea fowl | Yaoundé, Batouri, Maroua, Garoua, Ambam | Centre, East, Far North, North, South | I, IV, V | [43] |
|  | *Am. paulopunctatum* | Cattle, Warthog, Pig | Batouri, Akonolinga, Kribi | East, Centre, South | IV, V | [43] |
|  | *Am. variegatum* | Human, Cattle, Sheep, Goats, dog, Nile monitor, Four-toed hedgehog, Gazelle, Buffalo, Patas, Herisson, Grimm’s Cephalophe, Ourébi, Clapperton’s Francolin | Ndéré, Yaoundé, Douala, Buea, Wakwa, Dschang, Nkong-Ni, Massangam, Koutaba, Kouoptamo, Maroua, Meinganga, Mora, Fort-Foureau, Bocklé, Wakwa, Garoua, Mindif, Yagoua, Mora, Badjari, Lougguere, Obala, Edéa, Dumbo, Sarkimata, Meyomessala, Meyomessi, Sangmelima, Zoetele, Yoko, Vela Mbaï, Dang, Darang, Borongo, Maoui, Tello, Bambui, Nkwen, Mfonta, Limbé, Muyuka, Tiko, Bangourain, Foumban, Bangangte | Far North, North, Adamawa, west, Centre, Littoral, Southwest, Northwest, South, East | I, II, III, IV, V | [16,29,30,36,54,62] |
|  | *Am. tholloni* | Elephant | Kribi, Lolodorf, Edéa, Yaoundé, Ezéka, Fort-Foureau | South | IV | [43] |
|  | *Am. splendidum* | Cattle, Buffle (Syncerus coffer) | Bipindi, Mbam, Yaoundé, Mbalmayo, Ebolowa, Abong-Mbang, Evodoula, Bafia, Foumban, Ngaoundéré, Dschang, Djouma, Yoko | South, Centre, West, Adamawa | II, III, IV, V | [43] |
|  | *Am. cohaerens* | Cattle | Yaoundé | Centre | IV | [43] |

**Table S2:** *Hyalomma* ticks (8 species), associated hosts, collection locality, Region, and Agro-Ecological Zones

| **Ticks Genus** | **Tick species** | **Host** | **Locations** | **Region** | **AEZs** | **References** |
| --- | --- | --- | --- | --- | --- | --- |
| *Hyalomma* | *Hy. detritum* | Cattle (From Adamawa, North) | Yaoundé (slaughterhouse) | Centre | IV | [37] |
|  | *Hy. dromedarii* | Cattle (From Adamawa,) | Yaoundé (slaughterhouse) | Centre, North | IV, I | [16,37] |
|  | *Hy. excavatum* | Cattle | Nkong-Ni | Ouest | III | [27] |
|  | *Hy. impeltatum* | Buffon cob, Goats | Nord, Fort-Foureau, Dschang, Yaoundé | Far North, North, West, Centre | I, III, IV | [37,43] |
|  | *Hy. impressum* | Cattle | Yaoundé, Sakgmé, | Centre, Far North | I, IV | [43] |
|  | *Hy. nitidum* | Cattle, Red flanked duiker, Sheep Goats, Buffalo, Hare, Zebra | Lom Pangar, Garoua, Mindif (Kaélé), Yagoua, Badjari, Lougguere, Mora, Yaoundé, Obala, Edéa, Bambui, Nkwen, Mfonta, Yaoundé, Wakwa | East, Northwest, Adamawa, North, Far North, Centre | I, II, III, IV, V | [34,50,39,54,48,37,30] |
|  | *Hy. rufipes* | Cattle, Four-toed hedgehog, Pig, Phacochère, Horses, Buffalo, Hare, Zebra | Kaele, Yaoundé, Maroua, Fort-Foureau, Sagme, Wasa, Nkong-Ni, Dschang, Vela Mbaï, Dang, Darang, Wakwa, Borongo, Bambui, Nkwen, Mfonta, Bangourain, Foumban, Koutaba, Bangangte | Far North, Centre, Northwest, West | I, II III, IV | [43,34,50,39,54,48,96,16,37,30] |
|  | *Hy. truncatum* | Cattle, Sheep, Goats, Four-toed hedgehogs, Nile monitor, Buffalo, Warthog, Dog Horse, The Sagittarian Messenger, Buffalo cob, Antelope, Antelope horse, Cape Hare Herisson, Giraffe, Crested woodcock, Nile Rat | Kaélé, Ngaoundéré, Maroua, Wasa, Fort-Foureau, Tchevi, Guili, Bafia, Adamawa, Garoua, Yaoundé, Mbal-Mayo, Garoua, Mindif (Kaélé), Yagoua, Badjari, Lougguere and Mora, Dschang, Nkong-Ni, Vela Mbaï, Dang, Darang, Wakwa, Borongo, Bui, Donga-Mantung, Bangourain, Foumban, Koutaba, Bangangte | Far North, North, Adamawa, West, Centre, | I, II, III, IV, V | [16,30,34,37,43,48,50,53,96] |
|  | *Hyalomma* spp. | Cattle | Wakwa, Bambui, Jakiri, Weh, Tan | Northwest, Adamawa | II, III | [47] |

**Table S3:** *Rhipicephalus* ticks (26 species), associated hosts, collection locality, region, and Agro-Ecological Zones

| **Ticks Genus** | **Tick species** | **Host** | **Locations** | **Region** | **AEZs** | **References** |
| --- | --- | --- | --- | --- | --- | --- |
| *Rhipicephalus* | *R. annulatus* | Cattle, Sheep, Goats | Yaoundé, Bafia, Meiganga, Ngdéré, Fort-Foureau, Sagmé, Tchevi, Obala, Edéa, Sarkimata, Dschang, Vela Mbaï, Dang, Darang, Wakwa, Borongo, Bui, Donga-Mantung, Bambui, Nkwen, Mfonta, Bambili, Bangourain, Foumban, Koutaba, Bangangte | Far North, Adamawa, Centre, Littoral, West, Northwest, North | I, II, III, IV | [43,34,50,26,74,46,47,35,16,27,37,29] |
|  | *R. aurantiacus* | Cattle, Buffalo, Warthog | Molundu, Batouri, Bipindi | East, South | IV | [43] |
|  | *R. appendiculatus* | Cattle, | Ngaoundere, Wakwa, Maoui, Tello | Adamawa | II | [38] |
|  | *R. brevicoxtus* | - | Mbalmayo | Centre | IV | [43] |
|  | *R. boueti* | Cattle, Daman | Maroua | Far North | I | [43] |
|  | *R. cliffordi* | Cattle, Buffalo, Warthog | Yoko | Centre | IV | [43] |
|  | *R. camicasi* | Red flanked duiker | Lom Pangar | East | IV | [30] |
|  | *R. complanatus* | Warthog, Homme, Rodent | Batouri, Molundu, Yokadouma, kribi, Bafia, Akonolinga, Lomié, Mamfé, | East, South, Centre, Southwest | IV, V | [43] |
|  | *R. cuspidatus* | Cattle, Warthog, Erycterope, | Maroua, Fort-Foureau, Waza, Logorne-Birni | Far North, North | I | [43] |
|  | *R. decoloratus* | Cattle, Gazelle, Antilope, Bubale, Sheep, Goats, Horses | Maroua, Ngdéré, Fort-Foureau, Tchevi, Ngodeni, Bocklé, Garoua, Mindif (Kaélé), Yagoua, Badjari, Lougguere, Mora, Yaoundé, Obala, Edéa, Sarkimata, Nkong-Ni, Dschang, Vela Mbaï, Dang, Darang, Wakwa, Borongo, Bui, Donga-Mantung, Bambui, Nkwen, Mfonta, Bambili, Bangourain, Foumban, Koutaba, Bangangte, Banso, Fundong, Acha, Fongo-Tongo, Fokoué, | Far North, Adamawa, North, Centre, Littoral, Northwest, South, West | I, II, III, IV, V | [43,34,50,26,74,46,47,35,16,27,37] |
|  | *R. evertsi* | Cattle, Donkey, Horse, Rodent, Sheep and Goats | Maroua, Waza, Fort-Foureau, Ebolowa, Meyomessala, Meyomessi, Sangmelima, Zoetélé | Far North, East, South | I, IV, V | [43,52] |
|  | *R. geigyi* | Cattle, Sheep, Goats | Wakwa, Bambui, Jakiri, Weh, Tan, Nkong-Ni, Vela Mbaï, Dang, Darang, Wakwa, Borongo, Bui, Donga-Mantung | Northwest, Adamawa, West | II, III | [47,35,27,53] |
|  | *R. guihloni* | Cattle, African savanna hare, Four-toed hedgehog, Nile monitor, Caracal, Arabian bustard, Cat, Gazelle, korrigum, Herisson, Jabiru, African Marabou, Squirrel, Rodent | Lom Pangar, Maroua, Wasa, Fort-Foureau, Nkong-Ni | East, West | IV, III | [43,27,30] |
|  | *R. linnaei* | Nile monitor | Kaele | Far North | I | [30] |
|  | *R. longus* | Cattle, Dog, Lion, Warthog, Buffalo, | Yaoundé, Ntui, Saa, Bafia, Banganté, Batouri, Douala, Kribi, Dschang, Mbalmayo, Sagba, Foumban, Edéa, Nkong-Ni, Massangam, Koutaba, Kouoptamo | Centre, South, West, East, Littoral, Northwest | III, IV, V | [43,26] |
|  | *R. lunutatus* | Cattle, Goats, Warthog, Ourebi | Foumban, Ngdéré, Maroua, Fort-Foureau, Isora, Yaoundé Dschang, Nkong-Ni, Massangam, Kouoptamo, Koutaba, Bambui, Nkwen, Mfonta, Fokoué | Far North, North, Adamawa, West, Centre, Northwest | I, II, III, IV | [43,26,39,54,46,47,66,96,29,50] |
|  | *R. microplus* | Cattle, Four-toed hedgehogs, Nile monitor, Sheep | Kaele, Dschang, Nkong-Ni, Massangam, Koutaba, Kouoptamo, Ngaoundere, Wakwa, Maoui, Tello, Bangourain, Foumban, Bangangte , Yaoundé | Far North, West, Centre, Southwest, South | I, II, III, IV, III, V | [38,16,44,37,30,29] |
|  | *R. moucheti* | Cattle, Red flanked duikers, antelope, Monkeys, Four-toed hedgehogs, hares, Nile monitor, Patas | Lom Pangar, Maroua | East, Far North | IV, I | [30,43] |
|  | *R. mushamae* | Cattle, Rats, Nile monitor, Warthog, Civet, Buffalo Cob, Zorille commune, antiloppe cheval, Ecureiul fouisseur | Soramboum, Kaele, Garoua, Maroua, Tchevi, Fort-Foureau, Logone-Birni, Djamba, Dschang, Nkong-Ni | Far North, North, West | I, III | [43,27,29,30] |
|  | *R. pusillus* | Cattle | Ngaoundere | Adamawa | II | [16] |
|  | *R. sanguineus* s.l. | Cattle, Human, Dog, Buffalo Cob, Ourebi, Hare, Primate, Buffalo, Bird, Sheep, Goats, | Douala, Yaoundé, Mbalmayo, Dschang, Maroua, Fort-Foureau, Bafia, Foumban, Edèa, kribi, Garoua, Mora, Yabassi, Ebogho, Bocklé, Nkong-Ni, Meyomessala, Meyomessi, Sangmelima, Zoetélé, Vela Mbaï, Dang, Darang, Wakwa, Borongo, Limbé, Bangourain, Koutaba, Bangangté | Far North, North, Centre, Littoral, West, Southwest | I, II III, IV, V | [35,43,62,90,16,44,37,29] |
|  | *R. senegalensis* | Cattle, Pig, Warthog, Buffalo, Harnessed Guib | Yaoundé, Batouri, Akonolinga, Djoum | Centre, South, East | IV | [43] |
|  | *R. simpsoni* | Aulacaude | Yaoundé, Bafia | Centre | IV | [43] |
|  | *R. sulcatus* | Cattle, Gazelle, Buffon's Cobe, Cape Hare, Ourébie, Bird, Stork | Maroua, Ngdéré, Banganté, Dschang, Ebogho, Yaoundé, Mora, Bambui, Nkwen, Mfonta | Far North, Adamawa, Centre, West, Northwest | I, II, III, IV | [43,50,26,46,16] |
|  | *R. turanicus* | Cattle | Wakwa | Adamawa | II | [39] |
|  | *R. ziemanni* | Cattle, Cephalophus, Warthog, Sheep, Rodent, Leopard | Douala, Massamena, Lomié, Akonolinga, Yaoundé, Mbalmayo, Mamfé, Djoum, | East, Littoral, Centre, South | IV, V | [43] |
|  | *Rhipicephalus* spp. | Cattle | Wakwa, Bambui, Jakiri, Weh, Tan | Northwest, Adamawa | II, III | [54,47] |

**Table S4 :** *Haemaphysalis* ticks (11 species), associated hosts, collection locality, region and Agro-Ecological Zones

| **Ticks Genus** | **Tick species** | **Host** | **Locations** | **Région** | **AEZs** | **References** |
| --- | --- | --- | --- | --- | --- | --- |
| *Haemaphysalis* | *Ha. aciculifer* | Cattle, bucorve d'Abyssinie, | Koubadje, Yaoundé, Bambui, Nkwen, Mfonta | Adamawa, Centre, East, West, Northwest | I, IV | [43,50,26] |
|  | *Ha. camicasi* | Red flanked duikers, Nile monitors, Monkeys, Hares, Four-toed hedgehog, African rock pythons, Antelopes, Hedgehog (A. albiventris), *V. pallida*, Civet (*Civettictis civetta*), Cat (*F. silvestris*) | Ebolowa, Lom Pangar, Mboutwa, Maroua, Mindif | South, East, Far North | I, IV | [40,30] |
|  | *Ha. hoodi* | Blue-naped coucal, Francolin, rodent, Abyssinian bucorve, Human | Yaoundé, Foumban, Batouri, Koubadje, Nkozoa | Adamawa, Centre, East, West | II, III, IV | [43,24,65] |
|  | *Ha. houyi* | Cattle, Nile monitors, Red flanked duikers, Monkeys, African savanna hares, Four-toed hedgehogs, Antelopes, African rock python, Ecureuil | Kaélé, Lom Pangar, Tibati | Far North, Adamawa, East | I, II, IV | [30,43] |
|  | *Ha. leachi* | Cattle, White-bellied pangolins, African civets, Dog, Cat, Rodent, Panther, Fox, Wild cat, Mongoose, Tiger genet, Lion, Francolin, Sheep, Goats | Ebolowa, Yaoundé, Maroua, Mamfé, Logone-Birni, Foumban, Dschang, Bafia, Edea, Djoum, Lolodorf, Ebogho, Batouri, Yokadouma, Abong-MBang, Ntui, Saa, Mbalmayo, Ndikinimeki, Nkong-Ni, Vela Mbaï, Dang, Darang, Wakwa, Borongo, Bui, Donga-Mantung, Buea, Douala | Centre, North, West, Southwest | I, II, III, IV, V | [25,43,63,64,53,30,29] |
|  | *Ha. punctaleachi* | Civet (*Civettictis civetta*), Tiger genet (*Genata tigrina*) | Yaoundé, Obala, Molundu, Nanga-Eboko, Evodula, Nkomakak-Efoulan | Centre | IV | [33] |
|  | *Ha. moreli* | Cattle, Civet (*Civettictis civetta*), Genata sp. | Yaoundé, Obala, Edéa, Manfe, Efoulan, Nanga-Eboko, Evodula, | Centre, Littoral, Southwest | IV, V | [34,61] |
|  | *Ha. muhsamae* | Cattle, Mongoose, Genet, Hedgehog | Rei-Bouba, Maroua, Dschang, Nkong-Ni | Far North, North, West | I, III | [29,43] |
|  | *Ha. paraleachi* | Cattle | Dschang, Nkong-Ni | West | III | [29] |
|  | *Ha. parmata* | Cattle, Nile monitor, Antelope, Pig, Goat, Sheep, Civet, Genet, Duiker, Guinea fowl, Squirrel, Passerines | Ebolowa, Douala, Mont Bakossi, Buea, Yaoundé, Akonolinga, Bafia, Saa, Efoulan, Kribi, Ambam, Djoum, Lomié, Molundu, Ndikinimeki, Mamfé, Batouri | Centre, South, Southwest, Littoral, East | IV, V | [30,43] |
|  | *Ha. princeps* | Civet (*Civettictis civetta*), *V. pallida* | Mboutwa, Maroua | Far North | I | [40] |
|  | *Haemaphysalis* spp*.* | Dog | Buea, Douala | Sud-Ouest, Littoral | V | [62] |

**Table S5:** *Ixodes* ticks (7 species), associated hosts, collection locality, region and Agro-Ecological Zones

| **Ticks Genus** | **Tick species** | **Host** | **Locations** | **Région** | **AEZs** | **Reference** |
| --- | --- | --- | --- | --- | --- | --- |
| Ixodes | *I. aulacodi* | Aulacaude (*Tryonomys swinderianus*), Guinea fowl (*Numida meleagris*), Bird (*Prinia leucopogon*) | Yaoundé, Yokadouma, Abam, Bafia | Centre, East, | IV | [43] |
|  | *I. cumulatimpunctatus* | Cattle, Guinea fowl, Warthog, Mangoose | Yaoundé, Molundu, Lomié, Ambam, Akono, Bambili, Ndop, Mankon, Nkambé, Sagba | Far North, Northwest | I, III, | [43,50] |
|  | *I. moreli* | Antilope | Ebolowa | East | IV | [30] |
|  | *I. muniensis* | Panther, Duiker, Antelope | Manfé, Yokadouma, Djoum, Molundu, Mbalmayo, Nzolok | Southwest | V | [43] |
|  | *I. rageaui* | Human, Moustac (*Cercopithecus cephus*) | Yaoundé, Bafia, Evodoula | Centre, | IV | [43] |
|  | *I. rasus* | Cattle, White-bellied pangolins, Four-toed hedgehog, Warthog, Monkey, Water chevrotain, Panther, Dog, Nandinie, Pangolin, Duiker, Mangoose, Guinea fowl, Francolin, Passerines, Bongo, Bushpig, Antilope | Yaoundé, Lolodorf, Molundu, Ebolowa, Mandama, Mamfé, Basham, Efoulan, Ambam, Batouri, Kribi, Lomié, Mbalmayo, Nkong-Ni | North, Centre, East, South, Southwest, West | I, III, IV, V | [43,30,29] |
|  | *I. vanidicus* | Civet, Dog, Mangoose (*Atilax paludinosus*) | Yaoundé, Kampo, Mamfé, | Centre, Sud, Sud-Ouest | IV, V | [43] |

**Table S6 :** Other ticks species, associated hosts, collection locality, region and Agro-Ecological Zones

| **Ticks Genus** | **Tick species** | **Host** | **Locations** | **Région** | **AEZs** | **References** |
| --- | --- | --- | --- | --- | --- | --- |
| *Aponomma* | *A. arcanum* | Varan, | Douala, Edéa, Mamfé, | Littoral, Southwest | V | [43] |
|  | *A. flavomaculatum* | Varan, Python sebea | Karba-Manga, Nditi, Maroua, Ngouyanga, Sakdge, Bangui, | Far North, North, Adamawa, Centre | I, II, IV | [43] |
|  | *A. latum* | Python, Naja melanoleuca (Cobra) | Sangmélima, Maroua, Yaoundé, Ebolowa, Evodoula, Djouma, Rei Bouba | Far North, Centre, South | I, IV | [43] |
|  | *A. transversale* | Python sebae | Dibombari, Yaoundé | Centre, Littoral | IV, V | [30,43] |
|  | *A. exornatum* | Varan | Douala, Edéa, Ndiki, Yaoundé, Sakdgé | Littoral, Centre, North | I, IV, V | [43] |
| *Dermacentor* | *D. circumguttatus* | Elephant | Kribi, Lolodorf, Fort-Foureau, Bipindi, Barombi, Ezeka | Centre, South, Far North |  | [43] |
| *Argas* | *A. persicus* | Poultry | Maroua, Wasa | Far North | I | [43] |
|  | *A. arboreus* | Grey Pelican (*Pelecanus rufescens*), Pied Raven (*Corvus albus*), Crowned Crane (*Balearica pavonina*), Martial Eagle (*Polemaetus bellicotus*) | Maoura, | Far North | I | [43] |
| *Carios* | *C. vespertilionis* | Bats | Meiganga | Adamawa | II | [43] |
| *Ogadenus* | *O. brumpti* | Herisson | Wasa, Maroua | Far North | I | [43] |
